# Predictive value of the fibrinogen-to-high-density lipoprotein cholesterol ratio for sub-clinical diabetic peripheral neuropathy in type 2 diabetes mellitus

**DOI:** 10.3389/fnagi.2026.1772503

**Published:** 2026-04-20

**Authors:** Yanmei Wang, Fan Hu, Jie Li, Xiaoqing Lu, Yaoyao Shen, Jinhua Chen, Hong Zhang

**Affiliations:** 1 Department of Neurology, Jiangxi Provincial People’s Hospital, The First Affiliated Hospital of Nanchang Medical College, Nanchang, Jiangxi, China; 2 Department of Neurology, National Regional Center for Neurological Diseases, Xiangya Hospital, Central South University, Jiangxi, China

**Keywords:** fibrinogen, high density lipoprotein cholesterol, mellitus, sub-clinical diabetic peripheral neuropathy, type 2 diabetes

## Abstract

**Introduction:**

This study aims to investigate the predictive value of the fibrinogen-to-high-density lipoprotein cholesterol ratio (FHR) in relation to sub-clinical diabetic peripheral neuropathy (sDPN) in individuals diagnosed with type 2 diabetes mellitus (T2DM).

**Methods:**

A cohort of 281 patients with T2DM was admitted to the Neurology Department of Jiangxi Provincial People’s Hospital between January and December 2023. Within this population, 148 patients were diagnosed with sDPN. The clinical profiles, inflammatory biomarkers, and nerve conduction velocities or current perception thresholds (CPTs) were compared between the two distinct groups. A logistic regression analysis was performed to identify the risk factors for sDPN. The predictive performance of each factor was assessed using a receiver operating characteristic (ROC) curve analysis. The FHR was compared among three groups, which were based on the severity of peripheral neuropathy (PN).

**Results:**

Patients with sDPN exhibited significantly elevated levels of fibrinogen (FIB), fasting plasma glucose (FPG), hemoglobin A1c (HbA1c), the monocyte-to-lymphocyte ratio (MLR), and the platelet-to-lymphocyte ratio (PLR), along with diminished high-density lipoprotein cholesterol (HDL-C) levels, compared to patients without sDPN. Univariate and multivariate logistic regression analyses indicated that age, FIB levels, HbA1c, HDL-C, and MLR were significant risk factors that contributed to the onset of sDPN in T2DM patients. The ROC curve analysis indicated that the FHR had an area under the curve (AUC) of 65%. The optimal cutoff value for the FHR was 2.65, exhibiting a specificity of 62.4% and a sensitivity of 63.5%. The composite model incorporating the FHR demonstrated superior reclassification performance (net reclassification improvement (NRI) = 0.416, *p* = 0.001, 95% CI 0.180–0.650) and integrated discrimination improvement (IDI = 0.053, *p* < 0.001, 95% CI 0.001–0.015) compared to the basic model (age +HbA1c + MLR). Nonparametric test analysis showed significant differences in the FHR among the three groups. The more severe the PN, the higher the FHR.

**Conclusion:**

The FHR may serve as a valuable early diagnostic marker for sDPN in T2DM.

## Introduction

Diabetic peripheral neuropathy (DPN) is a prevalent chronic microvascular complication associated with T2DM, affecting nearly 50% of individuals with this condition (Mizukami and Kudoh, 2022). DPN is defined as the presence of symptoms or signs indicative of peripheral nerve dysfunction in T2DM patients, following the exclusion of other possible causes of peripheral neuropathy (PN). DPN is characterized by a gradual onset, insidious progression, and worsening symptomatology over time. Currently, the precise mechanisms that contribute to the onset of DPN remain elusive. It is predominantly associated with the activation of the polyol pathway, reduced levels of myo-inositol, metabolic inflammation, abnormalities in lipid metabolism, and oxidative stress (Alothman et al., 2018; Elafros et al., 2022). The prevalent symptoms of DPN include limb numbness and diminished sensation. Hyperglycemia and associated metabolic disturbances can compromise immune function and increase susceptibility to wound infections. If these infections are not promptly managed, the risk of developing diabetic foot ulcers can escalate considerably. This can potentially lead to ulcerative necrosis and severe complications such as amputation, septic shock, or even death. At present, the management of DPN primarily focuses on symptom alleviation. DPN is an irreversible condition that significantly affects the quality of life for diabetic individuals. Therefore, early detection of DPN is of paramount importance. Previous studies have demonstrated that high-density lipoprotein cholesterol (HDL-C) is recognized for its cardioprotective properties. It plays a crucial role in mitigating oxidative stress, exerting anti-inflammatory effects, and safeguarding vascular endothelial integrity (Iqbal et al., 2017). Recent investigations have increasingly focused on the role of neuroinflammation in the pathogenesis and progression of DPN. Systemic inflammation markers have been identified, including white blood cells (WBCs), C-reactive protein, and the systemic inflammatory response index. FIB is an acute-phase protein known to stimulate inflammatory cells to release pro-inflammatory cytokines, thereby exacerbating the inflammatory response (Wang et al., 2022; Zhang et al., 2019). Previous studies have primarily examined predictive factors related to peripheral neuropathy in DPN. However, research on patients in the subclinical stage is relatively limited. Our research focused on sDPN and explored relevant predictive indicators, providing a more comprehensive, integrated biomarker for the risk environment of microvascular and neuropathic complications in diabetic patients. Changes in FIB and HDL levels provide significant insights into the assessment of peripheral neuropathy in T2DM patients. The fibrinogen-to-HDL-C ratio (FHR) may reflect inflammatory changes associated with the disease or the initiation of multiple pathogenic mechanisms. It could serve as a predictive marker for the onset and progression of DPN. This study aims to evaluate the predictive value of the FHR in relation to sDPN in order to facilitate the early clinical identification of this condition.

## Materials and methods

### Research participants

This retrospective study included patients aged between 18 and 75 who were consecutively admitted to Jiangxi Provincial People’s Hospital and subsequently diagnosed with type 2 diabetes mellitus (T2DM) between January and December 2023. Data regarding general demographics were gathered. Detailed medical histories, including specific neurological symptoms, were documented based on patient self-reports and clinical observations. A comprehensive neurological examination was subsequently conducted. All participants underwent neuroelectrophysiological assessments, which included nerve conduction studies (NCSs) and current perception threshold (CPT) testing. sDPN was diagnosed using the unified DPN screening protocol developed by the Chinese Diabetes Society of the Chinese Medical Association in 2010, following the recommendations of the ADA in 2005 (Callaghan et al., 2012; Tesfaye and Selvarajah, 2012) and the Toronto Consensus (Dyck et al., 2011). The assessment involved a combination of symptom evaluation (using the Michigan Neuropathy Screening Instrument), a physical examination (including the 10-g monofilament test, assessment of pinprick sensation, measurement of vibration perception threshold with a 128-Hz tuning fork, and ankle reflex assessment), and nerve conduction studies. Patients were classified into two categories: sDPN (indicated by no symptoms and an abnormal CPT or NCS) and no DPN (characterized by no neuropathy symptoms, no sDPN signs, a normal NCS, and the presence of neuropathy symptoms alone or a single sDPN sign). T2DM patients had no other relevant complications besides retinal vascular disease and peripheral neuropathy.

Patients with a history of long-term or heavy alcohol consumption, occupational exposure to neurotoxins, PN injury, signs of radicular nerve irritation, cervical spondylosis, lumbar disc herniation, thyroid disease, or those taking painkillers for non-numbness-related pain relief were excluded from the study. In addition, individuals with vitamin B12 deficiency, leprosy, HIV infection, pregnancy or lactation, organic bladder dysfunction, liver or kidney dysfunction, cardiovascular diseases (including stroke, myocardial infarction, and atherosclerosis), or conditions that elevate inflammatory markers (such as tumors or pneumonia) were not included.

## Research methods

### Specimen collection

Blood samples were obtained from patients the morning after their admission. A complete blood count was conducted using the Beckman Coulter DxH 800 instrument. Fibrinogen (FIB) concentrations were measured using a Mindray C3100Vet fully automated coagulation analyzer. Biochemical indicators were analyzed using the Mindray BS-2800. The index was computed using the following formula:

SII = neutrophil count × platelet count/lymphocyte count (10^9/L)

MHR = monocyte count/high-density lipoprotein (HDL)

MLR = monocyte count/lymphocyte count

PLR = platelet count/lymphocyte count

TyG = ln [Fasting triglycerides (mg/dl) × fasting glucose (mg/dl)/2]

TGH = triglycerides/HDL

### Neuroelectrophysiological testing

Neuroelectrophysiological assessments were carried out by three distinct groups of physicians or technicians, with results kept confidential among them. Patients were instructed to recline comfortably and maintain a limb temperature of 32 °C. Electrophysiological evaluations were performed using the Keypoint.net electromyography and evoked potential system (Alpine BioMed ApS, Denmark).

Sensory and motor nerve conduction studies (NCSs): The median, ulnar, tibial, and common peroneal nerves of the bilateral limbs were evaluated. The amplitudes of sensory nerve action potentials and compound muscle action potentials were recorded, along with sensory conduction velocities, distal motor latencies, and motor conduction velocities.

CPT testing was conducted. Patients were seated in a quiet, private examination room, where a Neurometer® CPT device (Neurotron, USA) was utilized. Before the assessment, the procedure and the types of sensations to be experienced were thoroughly explained to the patients. A preliminary test was conducted to enhance their familiarity with the process for improved cooperation. Testing sites included the tips of the index fingers and the dorsal surfaces of the big toes. The stimulation electrodes consisted of two gold-plated disc electrodes. It delivered sine-wave electrical stimulation at frequencies of 2000 Hz, 250 Hz, and 5 Hz, targeting Aβ, Aδ, and C fibers, respectively. The stimulation intensity was gradually increased from 0.01 mA to 9.99 mA, and stimulation was immediately stopped once the patient perceived the stimulus. Each site and stimulation frequency was tested 3 to 4 times to determine the sensory threshold for that specific site and frequency. These parameters were verified through an automated testing cycle program.

### Statistical analysis

Statistical analysis was performed using SPSS version 20.0. The Kolmogorov–Smirnov test was employed to examine the normal distribution of continuous variables. For continuous variables exhibiting a normal distribution, the results were reported as means ± standard deviation. The independent samples *t*-test was applied for comparisons between two groups. Conversely, continuous variables that did not follow a normal distribution were expressed as medians and interquartile ranges (M, P25, P75). To compare two groups in this context, the Mann–Whitney U test was utilized, while the Kruskal–Wallis test was applied for analyses involving three groups. Categorical variables were presented as counts (percentages), with the chi-squared (χ^2^) test used for group comparisons. Univariate and multivariate logistic regression analyses were carried out to determine risk factors influencing the development of sDPN, with variable selection conducted using stepwise regression (specifying forward methods as necessary). Receiver operating characteristic (ROC) curves were generated to assess the predictive ability of the models. A *p*-value of < 0.05 was deemed statistically significant. To assess the additional predictive value, the net reclassification improvement (NRI) and integrated discrimination improvement (IDI) were calculated.

## Results

### Clinical characteristics

This study included a cohort of 281 diabetic individuals, of whom 148 had sDPN, and 133 were categorized as non-DPN. Compared to non-DPN patients, those with sDPN were older and showed elevated levels of several biomarkers, including disease duration, FIB, fasting plasma glucose (FPG), hemoglobin A1c (HbA1c), monocyte-to-lymphocyte ratio (MLR), platelet-to-lymphocyte ratio (PLR), monocyte-to-HDL-C ratio (MHR), triglyceride-glucose index (TyG), total cholesterol-to-HDL-C (TcH) ratio, triglyceride-to-HDL-C (TGH) ratio, creatinine (Cr), and fibrinogen-to-HDL-C ratio (FHR). Conversely, HDL-C levels were found to be diminished. No notable differences were identified regarding gender, body mass index (BMI), white blood cells (WBCs), total cholesterol (TC), triglycerides (TG), monocyte count, lymphocyte count, alanine aminotransferase (ALT), aspartate aminotransferase (AST), MHR, systemic immune-inflammation index (SII), neutrophil-to-lymphocyte ratio (NLR), and WBC-to-HDL-C ratio (WHR) (Table 1).

**Table 1:** Characteristics of the study population.

Variable	Non-DPN	sDPN	*p*-value
N (men/women)	133 (81:52)	148 (91:57)	0.828
Age (years)	55.24 ± 12.91	60.34 ± 11.09	**0.000**
Duration (years)	5.81 ± 5.54	7.34 ± 6.6	**0.038**
BMI (kg/m2)	24.61 ± 3.6	24.71 ± 4.51	0.834
WBC (L^−1^)	6.14 ± 1.72	6.41 ± 2.10	0.234
Monocytes (L^−1^)	0.47 ± 0.24	0.41 ± 0.16	0.569
Lymphocytes (L^−1^)	1.98 ± 0.68	1.87 ± 0.62	0.163
HbA1c (%)	8.27 ± 2.35	9.32 ± 2.77	**0.001**
FPG (mmol/L)	8.61 ± 3.83	11.56 ± 6.29	**0.000**
ALT (U/L)	30.04 ± 29.57	26.36 ± 15.06	0.19
AST(U/L)	24.18 ± 14.36	21.52 ± 8.31	0.060
Cr (μmol/L)	62.84 ± 18.22	68.39 ± 24.29	**0.035**
TC (mmol/L)	4.72 ± 1.05	6.25 ± 1.23	0.418
HDL-C (mmol/L)	1.07 ± 0.31	0.99 ± 0.27	**0.025**
FIB (g/L)	2.65 ± 0.69	2.98 ± 0.85	**0.001**
MHR	0.43 (0.29, 0.60)	0.46(0.36, 0.68)	0.42
MLR	0.22 (0.18, 0.28)	0.25 (0.20, 0.31)	**0.004**
PLR	101.13 (80.74, 134.33)	107.78(88.05, 143.81)	**0.029**
NLR	1.70 (1.36, 2.42)	1.83(1.53, 2.40)	0.235
SII	346.76 (264.35, 568.00)	379.33 (281.61, 529.16)	0.392
TyG	5.60 (5.10, 6.17)	5.93 (5.46, 6.45)	**0.020**
TCH	4.40(3.55, 5.13)	4.78(3.88, 5.78)	**0.030**
FHR	2.35 (1.90, 3.18)	3.05(2.32, 3.78)	**0.000**

The data are presented as means ± standard deviation (S. D.). For normally distributed data, values are shown as means ± S. D. and analyzed using Student’s t-test. In contrast, for data that did not follow a normal distribution, the results are expressed as medians with interquartile ranges (IQRs) and analyzed using nonparametric tests, specifically the Wilcoxon test. Categorical variables are presented as frequencies and proportions, with analyses performed using the chi-squared (χ^2^) test. Statistical significance is highlighted in bold (*p* < 0.05). ALT, alanine aminotransferase; AST, aspartate aminotransferase; WBC, white blood cells; BMI, body mass index; Cr, creatinine; FPG, fasting plasma glucose; HbA1c, hemoglobin A1c; HDL-C, high-density lipoprotein cholesterol; FIB, fibrinogen; NLR, neutrophil-to-lymphocyte ratio; TC, total cholesterol; MHR, monocyte-to-high-density lipoprotein ratio; PLR, platelet-to-lymphocyte ratio; MLR, monocyte-to-lymphocyte ratio. SII, Systemic Immune-Inflammation Index; TyG, triglyceride-glucose index; TCH, total cholesterol-to-HDL-C ratio; and FHR, FIB-to-HDL-C ratio.

For all ratio-based indices (MHR, FHR, TyG, MLR, PLR, NLR, SII, and TCH): unitless.

### Univariate and multivariate regression analyses

Variables with a significance level of *p* < 0.05 in the univariate regression analysis—specifically age, FHR, Cr, and MLR—were subsequently included in the multivariate regression analysis, while those without significant differences in the univariate analysis, including TCH, PLR, NLR, and duration, were not included in the multivariate regression analysis. The findings from this analysis indicated that the FHR, MLR, and age continued to serve as independent risk factors for sDPN (Table 2).

**Table 2:** FHR associated with the presence of sDPN in a logistic regression analysis (enter method).

Indicators	β (S. E)	OR (95% CI)	*p*-value
Age	0.040 (0.012)	1.041 (1.017–1.066)	**0.001**
HbA1c	0.154 (0.056)	1.166 (1.045–1.302)	**0.006**
MLR	0.388 (0.185)	1.521 (1.280–1.842)	**0.036**
Cr	0.002 (0.007)	1.002 (0.989–1.016)	0.732
FHR	0.536 (0.142)	1.709 (1.294–2.257)	**0.000**

Data are presented as regression coefficient (standard error), odds ratio (95% CI), and *p*-value. Logistic regression analysis (enter method) was used to evaluate the association between the FHR and sDPN after adjusting for other clinical and biochemical variables. Bold text indicates statistical significance (*p* < 0.05).

### Predictive performance of the FHR for sDPN

**Figure 1:**
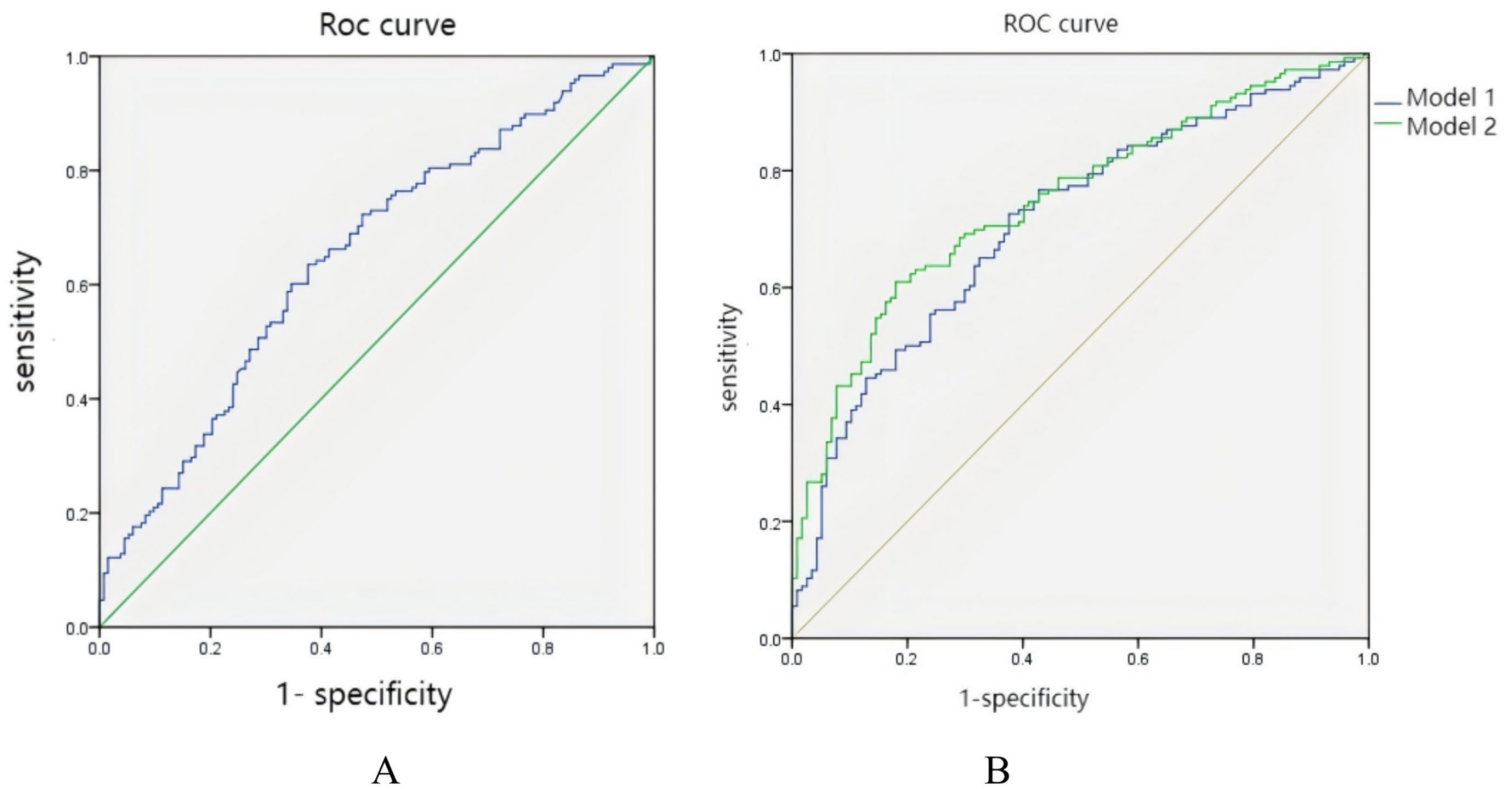
**(A)** ROC curve of the FHR for diagnosing sDPN. AUC = 0.65 (*p* < 0.001). The optimal cutoff value for the FHR was 2.65, exhibiting a specificity of 62.4% and a sensitivity of 63.5%. The curve demonstrates the modest but significant ability of the FHR to discriminate between patients with and without sDPN. **(B)** ROC curve for sDPN prediction by Model 1 and Model 2. The addition of the FHR demonstrated superior reclassification performance (NRI = 0.416, *p* = 0.001, 95% CI 0.180–0.650) and integrated discrimination improvement (IDI = 0.053, *p* < 0.001; 95% CI 0.001–0.015). FHR, FIB-to-HDL-C ratio; sDPN, sub-clinical diabetic peripheral neuropathy; AUC, area under the ROC curve. Model 1 includes age, MLR, and HbA1c, and Model 2adds the FHR to Model 1 (NRI = 0.416, IDI = 0.053, *p* < 0.05).

ROC curve analysis was performed for the independent predictive variable FHR. The area under the curve (AUC) was determined to be 65%, with an established optimal cutoff value of 2.65. The sensitivity and specificity recorded were 63.5 and 62.4%, respectively (Figure 1A). The FHR showed no significant AUC improvement when added to the basic model (age + HbA1c + MLR), increasing from 0.0.712 to 0.0.748 (*p* = 0.086, DeLong’s test) (Figure 1B). However, it demonstrated superior reclassification performance (NRI = 0.416, *p* = 0.001, 95% CI 0.180–0.650). For integrated discrimination improvement, substantial IDI gains were observed (IDI = 0.053, *p* < 0.001; 95% CI 0.001–0.015).

### Association between the PFR and sDPN

**Figure 2:**
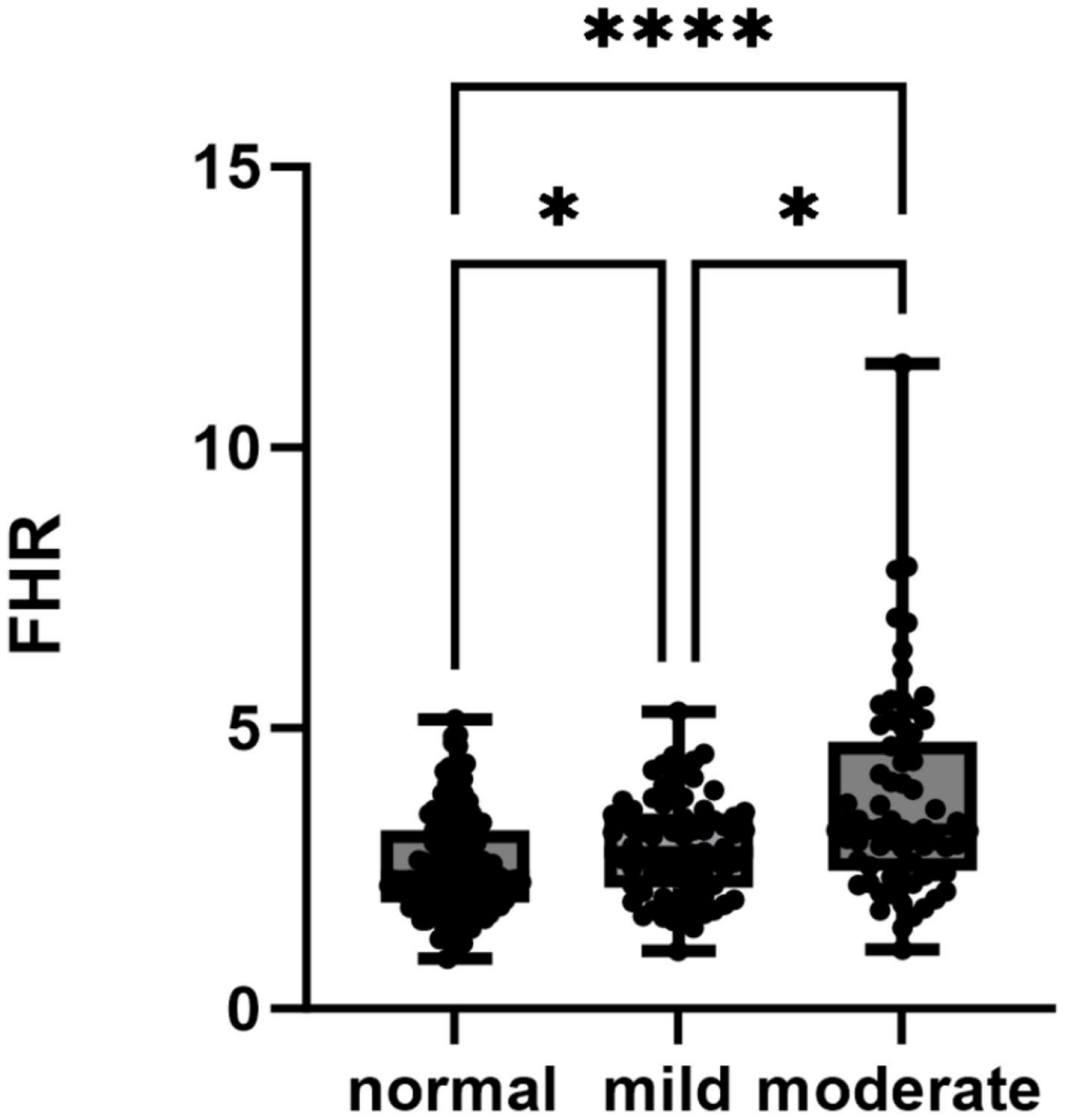
FHR levels across the different nerve damage groups were analyzed using a nonparametric test. **p < 0.05, **p < 0.01, ***p < 0.001, ****p < 0.0001. FHR, FIB-to-HDL-C ratio.

Early neuropathy primarily affects autonomic nerves and small fibers and gradually progresses to involve myelinated fibers and large fibers over time in patients with DM. The main manifestations of sDPN are mild-to-moderate nerve injury. NCSs are used to detect lesions in large fibers. Accordingly, patients exhibiting abnormal CPT results with normal NCS outcomes were classified as having mild neurological damage. Individuals presenting with abnormal NCS findings were categorized as the moderate group. Patients without peripheral neuropathy (PN) damage were assigned to the normal group. The analysis revealed significant variations in the FHR across the three groups, with a notable increase in the FHR correlating with the severity of PN (Figure 2).

## Discussion

DPN is a common complication characterized by significant morbidity and a gradually progressive course. Currently, there are no effective therapies available to reverse the condition. Early detection and management are important. This study aimed to evaluate the clinical features of sDPN and found that sDPN individuals tend to be older, have a longer duration of diabetes, increased HbA1c levels, and elevated biomarkers, such as FIB, MLR, PLR, Cr, and HDL-C, compared to non-DPN patients. Previous studies have identified several factors, including advanced age, extended diabetes duration, coagulation abnormalities, and elevated HbA1c, as being associated with the onset of DPN in T2DM patients (Cheng et al., 2022; Wang et al., 2023; Firouzabadi et al., 2023). These observations align with our findings. Older individuals are more susceptible to DPN, and their compromised peripheral circulation frequently leads to more severe complications at the time of diagnosis. Further analysis has shown that the MLR and FHR are independent predictive indicators for sDPN. In recent years, there has been a surge of research focusing on the role of neuroinflammation in the progression of DPN (Pop-Busui et al., 2016). Increasing evidence suggests that low-grade inflammation contributes to the pathogenesis of DPN. The NLR, PLR, and MLR have emerged as potential biomarkers for a variety of diseases and inflammatory conditions, including tumors, cardiovascular diseases, and other disorders (Vieceli Dalla Sega et al., 2022; Qiu et al., 2023). Our research further indicated that individuals with sDPN exhibited higher MLR and PLR levels relative to those without DPN. The MLR and FHR maintained their status as independent risk factors. This may reinforce the notion that inflammation is a significant contributor to the pathogenesis of PN.

Our study aimed to identify a novel, straightforward, and easily measurable biomarker for use before the onset of severe nerve damage. In our study, sDPN exhibited the highest FHR. A clinical investigation revealed that FIB levels in DPN individuals were elevated compared to non-DPN individuals in the T2DM cohort (Zhuang et al., 2022). It suggested that DPN patients experienced a more pronounced hypercoagulable condition. However, this study primarily focused on patients with DPN and did not investigate the prediction of sDPN. Our research further examined FIB levels in sDPN patients. It showed that FIB levels in sDPN patients were higher compared to non-DPN patients, but FIB was not an independent predictor of sDPN. In the context of diabetes, reduced HDL-C levels may contribute to endothelial dysfunction, oxidative stress, and the production of abnormal cytokines, all of which are linked to the onset and progression of DPN (Morton et al., 2012). Clinical investigations have substantiated this association, revealing that individuals with DPN exhibit significantly lower levels of HDL-C compared to non-DPN individuals (Christensen et al., 2020). Research involving animal models has shown that mice fed a high-fat diet experience demyelination of peripheral nerves (Xie et al., 2013). Although these observations arise from distinct experimental models, collectively they imply that dyslipidemia could hinder the formation of nerve myelin, thereby facilitating the onset and progression of DPN. We further explored HDL-C levels in patients with sDPN and found them to be lower compared to those without DPN; however, HDL-C did not emerge as an independent predictor of sDPN after adjustment. In addition, the FHR was identified as an independent predictor of sDPN. The AUC value of the FHR for predicting sDPN was 0.68. Although the predictive ability was relatively low, the model incorporating the FHR outperformed Model A in both classification accuracy and probability discriminative capacity. The AUC values of the two models showed no significant differences. It may imply that the FHR did not significantly enhance the overall discriminative ability of the model, but it had a positive impact on the accuracy of risk classification and predicted probabilities. This could be due to the FHR having higher predictive value within specific risk ranges or for specific subgroups of individuals, it may be that this effect was averaged out across the overall sample, or a small sample size leading to non-significant AUC differences. This may be attributed to the involvement of multiple pathogenic mechanisms in the early onset and development of DPN, including microcirculatory and neuroinflammatory pathways.

Elevated FIB levels and decreased HDL-C concentrations may be correlated with microcirculatory dysfunction and chronic low-grade inflammation, all of which contribute to an increased FHR. First, the unique pathophysiological rationale underlying the FHR’s association with sDPN may be related to microcirculatory metabolism. FIB is a hepatic protein integral to the coagulation process, and its plasma concentration significantly influences plasma viscosity. Microvascular alterations represent a key mechanism contributing to DPN. It may serve to facilitate the early detection and intervention of functional impairments in PN. In cases of poor glycemic control, elevated levels of insulin and glucose can lead to an increase in the secretion of plasminogen activator inhibitor-1 (PAI-1) by vascular endothelial cells. This results in an increased tendency for blood coagulation, stimulating FIB synthesis. Increased FIB concentrations can induce platelet aggregation, compromise the functionality of vascular endothelial cells, enhance coagulation potential, diminish fibrinolytic activity, elevate vascular permeability, and initiate various pathological changes, all of which further exacerbate microvascular injury (Zhu et al., 2024; Tracy and Dyck, 2008). The hypoxic conditions prevalent in the nerve microenvironment may intensify oxidative stress and inflammation, damaging stem cells (SCs) and neurons and ultimately leading to nerve injury (Gonçalves et al., 2017). Research has shown that the vascular architecture of the peroneal nerve in T2DM patients with DPN demonstrates significant alterations compared to non-DPN individuals. These changes include reduced expression of tight junction proteins, thickening of the microvascular basement membrane within nerve tissues, proliferation and swelling of vascular endothelial cells, and degeneration of pericytes. These modifications can lead to vascular constriction and impaired blood flow, resulting in ischemia and hypoxia of PN (Østergaard et al., 2015; Takeshita et al., 2020). If exacerbated over time, they can lead to degenerative atrophy of axons. This degeneration subsequently disrupts myelin function, leading to alterations in myelin structure, which further compromise nerve conduction capabilities. Research has demonstrated that among the various metabolic disturbances associated with T2DM, disorders of lipid metabolism are particularly common. Individuals with dyslipidemia frequently exhibit insulin resistance and persistent inflammation (Sears and Perry, 2015; Xin et al., 2023; Liu et al., 2023). In patients with T2DM, inadequate insulin secretion disrupts the equilibrium between lipid clearance and synthesis. This imbalance leads to a notable accumulation of lipids within the vascular walls, fostering the formation of microthrombi and hindering blood circulation. Altered lipid metabolism is a precursor to atherosclerosis, which subsequently induces modifications in vascular structure and hemodynamics. These alterations compromise the supply of nutrients to nerve microvessels, resulting in neuronal tissue damage and eventually contributing to the development of DPN (Rumora et al., 2022; Meir et al., 2024). Metabolic syndrome (MetS) can diminish the capacity for glucose uptake in the PN system. MetS is linked to analogous insulin resistance observed in muscular and adipose tissues. This condition leads to a depletion of energy reserves within axons, culminating in progressive nerve damage. Research has indicated that mice fed a high-fat diet develop MetS. It leads to energy deficits in elongated axons and induces neuropathological alterations, such as axonal degeneration and demyelination, as well as compromised axonal mitochondrial transport and biogenesis (Sajic et al., 2021).

Second, the pathogenic mechanisms may be closely related to neuroinflammatory factors. FIB is known to promote inflammatory processes. It can modulate the inflammatory response and the anti-inflammatory activities of macrophages. Specifically, FIB stimulates macrophages to adopt a pro-inflammatory phenotype, resulting in increased secretion of TNF-*α* and various other inflammatory cytokines, such as IL-6, MCP-1, MIG, MIP-1α, MIP-1β, and RANTES. These inflammatory responses have the potential to cause irreversible damage to neurons and glial cells (Hsieh et al., 2017). In addition, FIB is a biochemically unstable glycoprotein that can undergo non-enzymatic glycation due to structural alterations, ultimately leading to the generation of advanced glycation end-products (AGEs). These AGEs interact with their specific receptors, prompting the release of chemokines and pro-inflammatory markers, including nuclear factor kappa-light-chain-enhancer of activated B cells (NF-κB), TNF-α, and interleukins (Parwani and Mandal, 2023). Such events activate pathways that trigger inflammation, leading to damage of small blood vessels and dysfunction of glial cells, which may contribute to the loss of autonomic nerve neurons. The inhibition of FIB glycation and its structural modifications could potentially mitigate the progression of neuropathy (Chen et al., 2023). Research conducted by Schachtrup et al. showed that the binding of FIB to β3 integrin on neurons initiates EGFR phosphorylation, which subsequently hinders axonal growth (Ban et al., 2023). HDL-C is recognized as a protective factor for cardiovascular health, exhibiting multiple functions, including antioxidant, anti-inflammatory, and insulin resistance-reducing properties (Iqbal et al., 2017; Allard-Ratick et al., 2021; Rysz et al., 2020). HDL-C serves as a crucial lipid biomarker that is known to diminish in the presence of endothelial dysfunction and atherosclerosis. HDL-C effectively reduces the expression levels of certain endothelial adhesion molecules, including vascular cell adhesion molecule-1 (VCAM-1) and intercellular adhesion molecule-1 (ICAM-1), through various mechanisms such as the inhibition of oxidative stress and the modulation of signaling pathways. Therefore, HDL-C exhibits additional beneficial effects, including antioxidant, anti-inflammatory, and antiplatelet activities. It also plays a role in inhibiting monocyte activity and the polarization of macrophages by reducing IκB kinase activity, which, in turn, leads to a suppression of nuclear factor kappa-light-chain-enhancer of activated B cells (NF-κB) activity and a reduction in the inflammatory response (Rysz et al., 2020; Canpolat et al., 2016; Uittenbogaard et al., 2000). The findings indicated that patients experiencing severe PN impairment exhibited markedly elevated FHR levels. This suggests that metabolic irregularities, microcirculatory issues, and persistent low-grade inflammation, coupled with diminished anti-inflammatory and antioxidant capabilities, are critical factors in the development of DPN. Consequently, an elevated FHR may indicate a higher risk of developing DPN.

This study has certain limitations. Primarily, it used a cross-sectional design, and while the observed biomarkers demonstrated significant associations, such a design precludes definitive conclusions regarding causative relationships. The relatively small sample size may introduce potential bias, and the retrospective, single-center design may limit generalizability to broader populations. However, the cohort of patients with sDPN predominantly consisted of uncomplicated cases with few comorbid conditions, which strengthens the credibility of the results. The use of a consecutive sampling method during the study period helped minimize selection bias as much as possible within the retrospective framework.

In conclusion, the earliest stages of PN may be asymptomatic. The FHR has been identified as an independent risk factor for sDPN and shows potential for outcome prediction. Subsequent research endeavors will encompass multicenter prospective validation aimed at confirming the predictive significance of these biomarkers in larger and more varied populations. The establishment of a standardized system for ongoing monitoring and the creation of a dynamic warning model are essential. These initiatives will offer vital support for clinical risk assessment and the implementation of early interventions.

## Data Availability

The original contributions presented in the study are included in the article/supplementary material, further inquiries can be directed to the corresponding author.
